# Physiological and Psychological Effects of Viewing Urban Parks in Different Seasons in Adults

**DOI:** 10.3390/ijerph16214279

**Published:** 2019-11-04

**Authors:** Prita Indah Pratiwi, Qiongying Xiang, Katsunori Furuya

**Affiliations:** 1 Department of Landscape Architecture, Faculty of Agriculture, Bogor Agricultural University, Jalan Raya Dramaga, Bogor 16680, Indonesia; pritaindahpratiwi@ymail.com; 2 Department of Environmental Science and Landscape Architecture, Graduate School of Horticulture, Chiba University, 648 Matsudo, Matsudo-shi, Chiba 271-8510, Japan; xiang.q@chiba-u.jp

**Keywords:** blood pressure, heart rate, park therapy, seated viewing, profile of mood states, state-trait anxiety inventory, urban park

## Abstract

Although the health benefits of urban parks have gained significant attention, the effects of viewing park landscape on older adult residents have not commanded much research. This study aimed to investigate the physiological and psychological effects of viewing cherry blossoms in spring and fresh greenery in early summer at urban parks. The experiments were conducted in two viewing spots in the same park in Japan during different seasons, and included 12 participants in both spring (mean age, 66.4 ± 10.5 years) and summer (mean age, 65.75 ± 10.1 years). Participants sat and viewed an urban park and city landscape for 11–15 min. Blood pressure was measured before and after the seated viewing while heart rate was measured continuously. Then, they evaluated the psychological responses using the Profile of Mood States and State-Trait Anxiety Inventory. Viewing cherry blossoms and fresh greenery in urban parks led to lower blood pressure in spring and early summer than viewing city areas in spring and early summer. Moreover, the score of vigor–activity was significantly higher; tension–anxiety was significantly lower in spring; and the state-anxiety level was significantly lower in early summer. The results of this study suggest that viewing urban parks results in physiological and psychological relaxation.

## 1. Introduction

Aging and population growth have led to increasing cases of atherosclerotic vascular disease worldwide. As of 2015, almost 26.6% of Japan’s population was over the age of 65, exceeding Sweden (19.9%) and Italy (22.4%). This indicates that Japan’s aging population is progressing rapidly when compared to European countries [1]. In 2050, 37.7% of the Japanese population will be over the age of 65. Since the 20th century, strokes have been found to be highest cause of death in Japan. The main trigger for strokes is raised blood pressure or hypertension [2]. Turning one’s attention to forest bathing as a natural and low-cost activity is believed to alleviate stress-related diseases through plant-derived physiological relaxation [3]. This approach aims at “preventative medical effects” that induce physiological relaxation and improve immune functions to prevent diseases [4,5,6,7,8,9,10].

Studies demonstrated how brief walks in parks and forest environments reduced stress states and stimulated physiological and psychological relaxation. Studies on healthy young adults exhibited that brief walks in park and forest environments could reduce blood pressure [11,12], pulse rate [11], heart rate [13,14,15], and increase the natural logarithm of the high-frequency component of heart rate variability [13]. Forest and park therapy generated a significant increase in parasympathetic nervous activity and a significant decrease in sympathetic nervous activity [6,9]. Walking in urban parks leads to vigor [7,8,9], comfort, calmness, and accordance with nature [6], and significant decreases in tension, anger, fatigue, depression, confusion, and anxiety [7,8,9].

Several studies have investigated the effects of forest views in inducing physiological relaxation and reducing stress states in healthy young populations. The effect of viewing an urban forest landscape resulted in lower values in salivary cortisol concentration, pulse rate, and diastolic blood pressure in young males when compared with viewing urban landscapes [5]. Moreover, viewing a forest for 10–15 min significantly increased parasympathetic nervous activity and significantly decreased the heart rate of middle-aged hypertensive men [12]. This forest therapy method improved mood, heightened the positive impact, induced a feeling of subjective restoration and vitality for young male university students [16], and led to lower anxiety levels for young women [17]. The increasing scientific evidence on physiological relaxation improves human immune function recovery, prevents illnesses, and maintains and promotes health through exposure to nature [14,15].

Not all urban inhabitants have access to a natural forest. However, most cities and urban areas have a pocket of nature, such as a block park, neighborhood park, urban park, or urban forest, where there are plants that offer a relaxation benefit to people. Parks are valuable natural environments within a city. Most citizens across all ages have access to their preferred nearby parks [18]. In a study of park and garden landscape, Hofmann et al. described that gardening as model activity was an effective means of mitigating the harmful effects of chronic stress among Swiss citizens [19]. Ng et al. suggested that horticultural therapy could potentially be useful for reducing inflammation and protecting neuronal functions for healthy Asian elderly adults [20]. Xie et al. enforced the role of parks in supporting healthy aging, finding that older adults with best access to parks experienced lower odds of cardio-cerebral vascular diseases, joint diseases, and endocrine diseases than other citizens with the least access to the parks [21]. Additionally, the identified essential barriers to physical activity and park use (e.g., busy activity, lack of social support, weather-related concerns, and the fear of injuring oneself) will inform the design of a Park Prescription intervention in promoting physical activity, park use, as well as physical and mental well-being [22].

In a study of real-time park therapy, Igarashi et al. described that viewing a kiwifruit orchard landscape for 10 min resulted in induced physiological and psychological relaxation, such as a significant increase in the parasympathetic nerve activity; a significant decrease in heart rate; a significant increase in comfortable, relaxed, and natural feelings; and significant improvements in mood states for adult females [10]. Viewing a hospital rooftop forest for 12 min led to autonomic sensitivity to the forest’s natural elements and sufficiently relaxed older female patients [23]. Viewing a Japanese garden for 15 min reduced heart rate and improved the behavioral system in Japanese patients with dementia [24]. In another cross-cultural study, viewing different garden styles (e.g., Japanese garden, architectural garden, and landscape garden) induced psychological, emotional, and healing values among young Canadian and Japanese university students. Parasympathetic nervous activity increased in viewing the landscape garden, as the most natural-looking [25]. Most of the evidence-based studies have shown that the physiological and psychological benefits of walking or viewing in a forest and park landscape with varied environmental factors, including the type of natural environment, landscape design, as well as cultural differences, play a role in human interaction with the landscape. However, it might be difficult for middle-aged and elderly residents to visit natural forests due to lack of mobility, opportunity, and time. The effect of different seasons of park therapy in viewing urban parks—in this case, viewing cherry blossoms as the significant flower in Japan and fresh greenery in nearby urban parks—on middle-aged and elderly residents has been rarely investigated.

In Japan, a large part of the country is populated by forest, in which trees are particularly revered. Therefore, Japan’s forest area represents the relationship of “man in harmony with nature” [26]. We developed an evaluation of the physiological and psychological effects of a viewing experiment in spring and summer; the city’s prevailing climate is a major factor in the intensive use of the park. There is a seasonal visitor pattern in peak season, which is higher than the beginning and the end of the season [27]. Participants viewed Yoshino cherry (*Prunus yedoensis*) in spring and Katsura tree (*Cercidiphyllum japonicum*) in early summer in a large urban park in Matsudo, Chiba Prefecture, Japan. Yoshino cherry has been the most widely planted cherry species since the late 19th century [28], whereas Katsura tree is an indigenous shade deciduous tree which is now only sporadically found in China and Japan [29]. Matsudo city is a beautiful city suburb of Tokyo. A natural environment such as the Forest and Park for the 21st Century (FPC) within the urbanized city still exists, and is well managed every season.

This study aimed to clarify (1) the physiological relaxation effects and (2) the psychological relaxation effects of remaining sedentary while viewing cherry blossoms in spring and fresh greenery in early summer. The former were examined by measuring blood pressure and heart rate, whereas the latter were evaluated by assessing the Profile of Mood States (POMS) and State-Trait Anxiety Inventory (STAI). The general hypothesis was that there would be a significant difference in the psychological and physiological effects of seated viewing in an urban park and in a city area, as well as before and after the seated viewing.

## 2. Materials and Methods

### 2.1. Experimental Sites

**Figure 1:**
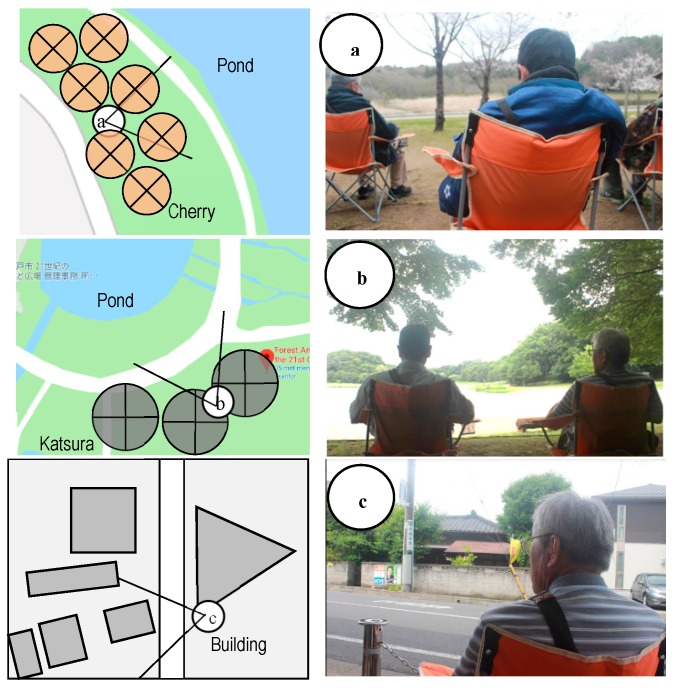
Schematic and photographs displaying the seated-viewing scenes: (**a**) urban park site in spring, (**b**) urban park site in early summer, and (**c**) city area site.

A field experiment was conducted from March to June 2019 in the FPC, Matsudo, Chiba Prefecture, Japan. Route 6 of Mabashihigashi, Matsudo, Chiba Prefecture was the urban area selected as the control site. The distance between the forest park and urban area was 2.8 km by car. The selection criteria for the urban park and city area were 1) safety, 2) well-maintained park or forest area, and 3) city areas located near downtown [17,30]. The FPC is large, spanning an area of 50.5 hectares with six types of sightseeing routes of natural landscape elements like ponds, farms and paddy fields, lawns, forests, wild grass gardens, and flower gardens. There were two viewing spots with the view of cherry blossoms in the foreground, pond and forest in the middle ground in spring, as well as fresh greenery (Katsura tree) in the foreground and pond and forest in the middle ground in early summer (Figure 1). The selection criteria for viewing spots included 1) shady refuge and 2) significant trees in the surroundings. The average temperature and relative humidity of the urban park and city area in spring and early summer are presented in Table 1.

### 2.2. Participants

Six middle-aged females and six males (mean age, 66.4 ± 10.5 years) participated in the spring experiment, and seven females and five males participated in the early summer experiment (mean age, 65.8 ± 10.1 years) (Table 2). A snowball sampling method was used to select key informants who were in close vicinity to the FPC. We asked two key informants, both male and female. Interviews were conducted to ensure the experiment day and participation. The key informants collected the participants living in Tokiwadaira, Mabashi, and Koganehara Districts. Study aims and procedures and eligibility criteria for participants in the form of flyers and guidance handouts were delivered to the key informants. The participants’ eligibility criteria were: 1) middle-aged and elderly over the age of 40, and 2) they were not taking blood pressure or heart rate medication. A total of 12 subjects per set of the experiment were sufficient to obtain significant information [17,30]. Additionally, the results of previous physiological studies have indicated that a sample size of 9–19 participants in the forest therapy experiment is sufficient to draw significant results [11,12,31]. Finally, 12 participants living in Tokiwadaira, Mabashi, and Koganehara Districts for each season were selected. Respondents were briefed on the study’s aims and procedures, and informed consent was obtained before the experiment. Alcohol, tobacco, caffeine, and food consumption were prohibited during the experiment, as was talking to other participants. The study was approved by the Ethics Committee of the Center for Environment, Health, and Field Sciences, Chiba University, Japan (Receipt code number: 18-06).

### 2.3. Experimental Design

**Figure 2:**
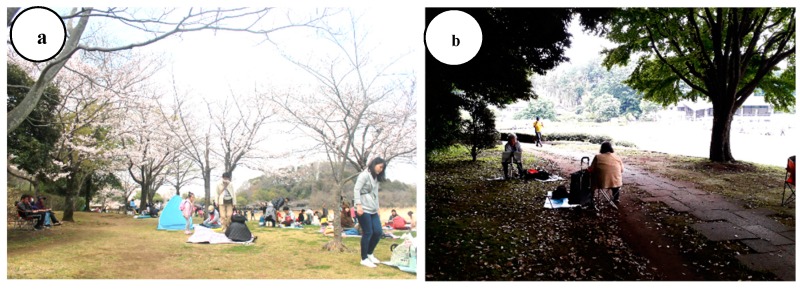
Seated-viewing experiment site in Forest and Park for the 21st Century: (**a**) cherry-blossom-viewing in spring; (**b**) leisure activities in summer.

To eliminate the order effects, 12 participants were randomly divided into two groups in a day. One group consisted of 2–4 participants in a day. Each group went to the experimental viewing spot in the park or city area in the morning. If the number of participants in a day was less than four people, there would be only one group that participated in a day. At the beginning of the seated-viewing experiment, participants received guided orientations and completed questionnaires; their blood pressure was measured, and the monitoring of their heart rates began. On the day of the experiment, each participant viewed the park or city area in the morning for 11 min in spring and 15 min in early summer (Table 3). Participants returned to the waiting room and completed questionnaires; their blood pressure was measured, and the monitoring of their heart rates stopped. They ate lunch containing the same number of calories and rested for 30 min. The experiment was repeated in opposite sites. Participants viewed the shading of Yoshino-cherry (*Prunus yedoensis*) in spring and of Katsura tree (*Cercidiphyllum japonicum*) in early summer (Figure 2). They viewed the landscape of Sendabori pond and forest as middle ground on the designated viewpoint.

### 2.4. Physiological and Psychological Indices

Heart rate was measured as a physiological response using a heart rate sensor (MyBeat WHS-3, Union Tool, Tokyo, Japan), whereas blood pressure was measured using a digital sphygmomanometer (Omron HEM-1021, Omron Corp., Kyoto, Japan). Two psychological scales, namely, the shortened Japanese version of the Profile of Mood States (POMS) and the State-Trait Anxiety Inventory (STAI), were delivered before and after the experiment. POMS, which was used to evaluate psychological responses to park viewing, comprised 35 adjectives, following six subscales: “anger–hostility” (A–H), “confusion–bewilderment” (C–B), “depression–dejection” (D–D), “fatigue–inertia” (F–I), tension–anxiety” (T–A), and “vigor–activity” (V–A). The total mood disturbance (TMD) score was calculated by combining A-H + C–B + D–D + F–I + T–A – V–A. A high TMD score indicated unfavorable psychological state [32,33]. As this study aimed to examine participants’ levels of anxiety influenced by park and forest environments, a Japanese version of the State-Anxiety part of STAI was used to measure state anxiety. State anxiety comprises 20 adjectives [34,35].

### 2.5. Statistical Analysis

Physiological and psychological data from 12 participants were analyzed. A paired *t*-test was used to compare mean physiological indices between the urban park and the city area, as well as before and after viewing in the urban park in each season. The Wilcoxon signed-rank test was applied to examine differences in reported psychological indices. All statistical analyses were performed using SPSS 22.0 (IBM Corporation, Armonk, NY, USA).

## 3. Results

### 3.1. Participant Characteristics

The mean age of the participants was 66.4 ± 10.5 in spring and 66.8 ± 10.1 in summer. The composition of gender was almost the same: 50% of males and 50% females in spring, 41.7% males, and 58.3% females in summer. Most of the participants (*N* = 24) were retired (70.83%), while the remaining participants were employed. Participants’ education background varied from senior high school, to university, and others. Half of the participants (*N* = 24) had an income of less than JPY 150,000/month (50%). Most of the participants did not smoke (91.7%) and had sleeping time less than 7 h (66.67%); nearly half of them did not drink alcohol (54.1%). They engaged in sports activity six times and shinrin-yoku six times in a month. About 58.3% (*N* = 24) had a medium level of social attachment to their neighborhood, with two times of participation in the community in a month.

### 3.2. Assessment of the Reliability of Physiological and Psychological Indices

Table 4 shows the internal consistencies (Cronbach’s alphas) of physiological and psychological indices among twelve subjects in spring and summer. The alpha reliability of physiological indices of heart rate in spring and summer was 0.815 and 0.824; for blood pressure it was 0.931 and 0.781, respectively. While the alpha reliability of the psychological response of the POMS score in spring and summer was 0.71 and 0.896, it was 0.896 and 0.933 for the STAI score, respectively. The results show that POMS and STAI had high internal consistency, while heart rate and blood pressure measures had reasonably good internal consistency. Therefore, all physiological and psychological indices had acceptable validity and reliability for this study.

### 3.3. Physiological Effects

**Figure 3:**
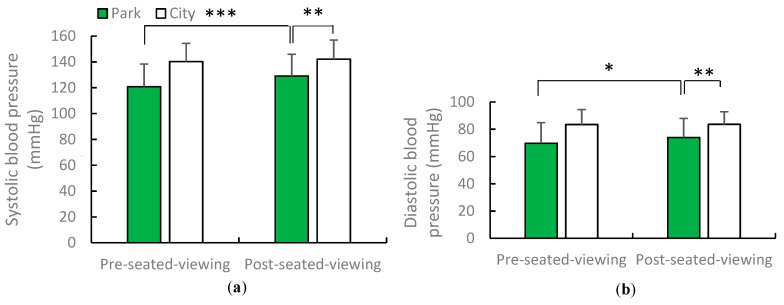
The average of blood pressure after urban park and city views in spring: **a**) systolic blood pressure and **b**) diastolic blood pressure. *N* = 12, mean ± standard deviation. * *p* < 0.05, ** *p* < 0.01, *** *p* < 0.001, determined by the paired *t*-test (one-sided).

**Figure 4:**
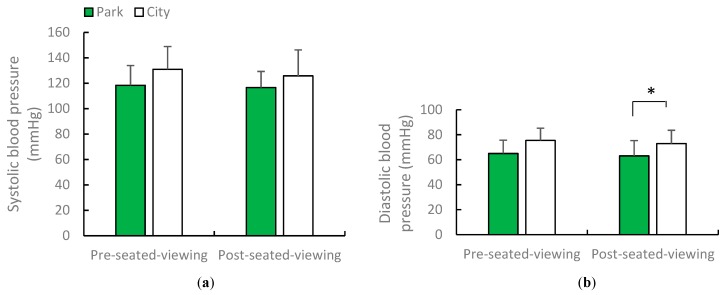
Average systolic and diastolic blood pressure after urban park and city views in early summer. *N* = 12, mean ± standard deviation. * *p* < 0.05, determined by paired *t*-test (one-sided).

**Figure 5:**
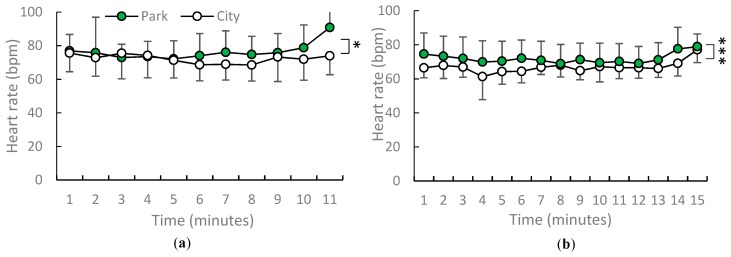
Average one-minute heart rate during urban park viewing in the: (**a**) spring and (**b**) early summer. *N* = 12, mean ± standard deviation. * *p* < 0.05, *** *p* < 0.001, determined by paired *t*-test (one-sided).

Compared with seated viewing in the city, seated viewing in the urban park was found to result in lower blood pressure but a higher heart rate. The magnitude of health benefits of viewing in the urban park on adults differed by season. The mean blood pressure was lower in the urban park (129.1/76 mmHg; systolic blood pressure: *p* = 0.0017; diastolic blood pressure: *p* = 0.0044) than in the city (142.1/83.6 mmHg). A paired *t*-test showed that the mean values of systolic and diastolic blood pressure in spring increased post-viewing compared with pre-viewing (*p* = 0.0005; *p* = 0.0405). In early summer, blood pressure was lower after viewing in the urban park compared with the city (post-urban park viewing: 116.7/63.1 mmHg; post city area viewing: 125.8/72.9 mmHg). The only significant difference in diastolic blood pressure was noted in the two environments in early summer (*p* = 0.0125). Figure 3 shows average systolic and diastolic blood pressure after the urban park and city area viewing in spring. Figure 4 shows average diastolic blood pressure after the urban park and city area viewing in early summer. In spring and early summer, the mean heart rates were higher when viewing in the urban park than those when viewing in the city (heart rate in spring urban park viewing was 76.52 bpm, city area viewing was 72.25 bpm; heart rate in summer urban park viewing was 71.97 bpm, city area viewing was 66.89 bpm; *p* = 0.011, *p* = 0.000). Figure 5 depicts the one-minute average heart rate during the urban park viewing in spring and early summer.

### 3.4. Psychological Effects

**Figure 6:**
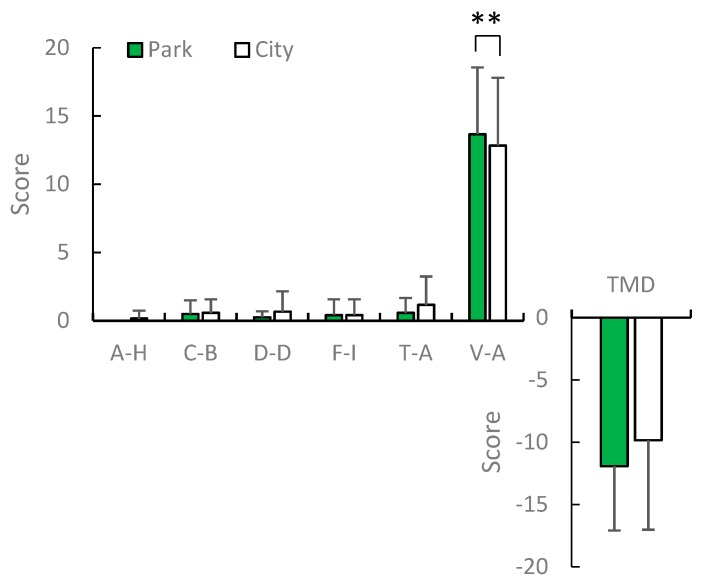
POMS scores after viewing in the urban park and city area in spring. A–H: anger–hostility; C–B: confusion–bewilderment; D–D: depression–dejection; F–I: fatigue–inertia; T–A: tension–anxiety; V–A: vigor–activity. *N* = 12, mean ± standard deviation. ** *p* < 0.01, determined by the Wilcoxon signed-rank test (one-sided).

**Figure 7:**
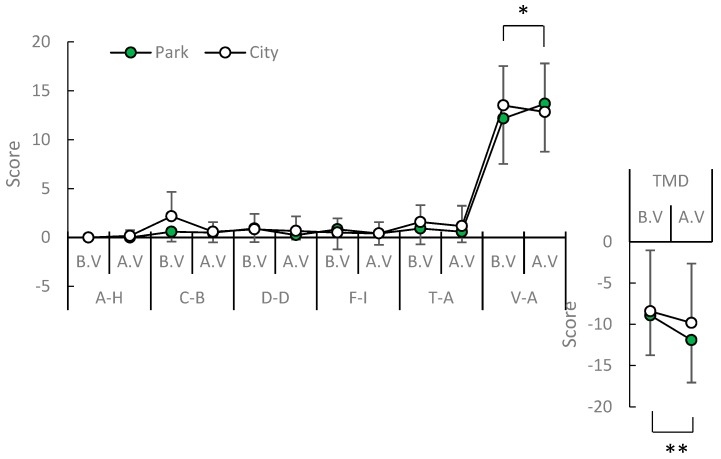
Comparison of POMS score pre and post-view in the urban park in the spring. A–H: anger–hostility; C–B: confusion–bewilderment; D–D: depression–dejection; F–I: fatigue–inertia; T–A: tension–anxiety; V–A: vigor–activity. *N* = 12, mean ± standard deviation. * *p* < 0.05, ** *p* < 0.01, determined by the Wilcoxon signed-rank test (one-sided).

**Figure 8:**
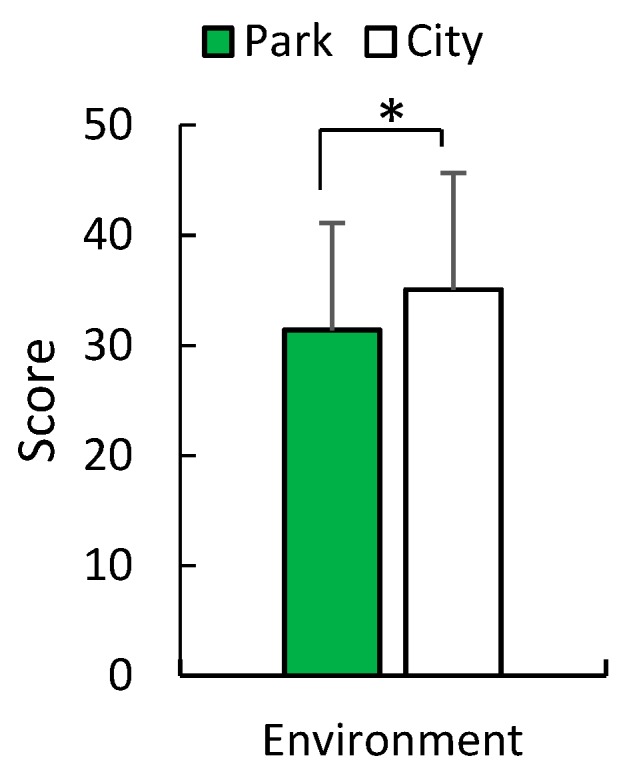
STAI score after seated-viewing in an urban park and the city in early summer. *N* = 12, mean ± standard deviation. * *p* < 0.05, determined by the Wilcoxon signed-rank test (one-sided).

In spring, a significant elevation of mood was only detected in the POMS test in the score for positive mood state “vigor–activity” (*p* = 0.001; Figure 6). The mean values of positive mood state “vigor–activity” increased in post-viewing compared with pre-viewing in an urban park (V–A: *p* = 0.023), whereas those of total mood disturbance decreased (TMD: *p* = 0.0045; Figure 7). There was no significant difference in the change in mood state in early summer in the two environments between post- and pre-viewing. According to the STAI, middle-aged residents exhibited a greater reduction in anxiety levels after viewing in the urban park compared with the city area in summer (*p* = 0.0135; Figure 8). The mean values of anxiety level decreased in post-viewing compared with pre-viewing in an urban park (*p* = 0.0085). There was no significant difference in anxiety level in the two environments between post- and pre-viewing in spring.

## 4. Discussion

This study clarified the physiological and psychological effects of viewing in urban parks during different seasons among middle-aged and older adult residents. Findings exhibited lower blood pressure and higher heart rate in 11-min seated viewing in an urban park in spring and 15-min seated viewing in early summer. Positive mood state (e.g., vigor–activity) was significantly higher in spring, and anxiety level was significantly lower in the urban park when compared with the city in early summer. Compared with pre-viewing in the urban park, a significant increase in positive mood state (e.g., vigor–activity) and significant decreases in total mood disturbance in spring and anxiety level were observed post-viewing in early summer.

Both systolic and diastolic blood pressure were lower during a brief viewing session in the urban park compared with those in the city area in spring and early summer. Various studies have reported a significant decrease in blood pressure and heart rate in park and forest environments [5,11,12,13,14,15,36]. The results of blood pressure responses were in line with those from previous studies, which investigated the physiological responses to urban park seated viewing. However, the heart rate response was contrary to previous studies, and indicated a higher rate after urban park viewing. Ikei et al. found that looking at blooming flowers—which is also called flower therapy—decreased heart rate and blood pressure [37]. By contrast, the higher heart rate after urban park viewing and lower blood pressure post-viewing in the urban park as compared to pre-viewing in spring might have been caused by the low temperature and the presence of cherry blossoms. Although the higher heart rate after urban park viewing in summer might be caused by water landscape, viewing cherry blossoms in spring significantly increases activity in the prefrontal area. Igarashi et al. described that the blooming Yoshino-cherry and human activity could have affected the activity of the prefrontal cortex, which was marked by the increased oxy-Hb concentrations in the prefrontal cortex [38]. This study revealed that people felt excited when viewing cherry blossoms [39]. In early summer, water bodies have a relative cooling impact on the evaporative procedure. Hence, evaporative cooling might be among the causes of passive cooling for the surroundings [40]. The existence of a water feature can serve as an attractor and confounding variable [41] and provide restorative benefits and stress reduction to garden users [42]. Both spring and early summer scenes containing water were associated with a significant positive effect and high perceived restorativeness [43]. Viewing in the urban park was more effective in blood pressure reduction than viewing in the city [6,11,12]. Decreased blood pressure post-viewing in the urban park compared with pre-viewing was observed in early summer, but no significant differences were detected. The environmental stimuli in summer, such as exposure to the sun and mosquitos in summer, other people passing by, and sudden activity and noise, might have influenced the blood pressure measurement [44].

We discovered that positive mood state such as “vigor–activity” increased in the urban park in spring, although the anxiety level declined in the urban park in early summer. Significant differences in “vigor–activity” and total mood disturbance were found post-viewing compared with pre-viewing in the urban park in spring. Various park therapy studies have reported a significantly decreased negative mood and anxiety level, as well as significantly enhanced mood after viewing urban parks [5,10,38]. Participants experienced more than one stimulation in spring compared with early summer, such as viewing cherry blossoms, the pond, and people’s activities under cherry blossoms in close-up view, and the forest in the distance. Increased stimulation led to higher positive moods through park therapy programs. These results are partly consistent with previous findings [44,45,46]. The magnitude of physiological and psychological benefits of the park through park therapy programs such as seated viewing were affected by temperature, exposure to the sun, and significant natural elements such as flowers, water bodies, and the coverage of greenery in the urban park. The present study demonstrated that among middle-aged and elderly residents, brief seated viewing of cherry blossoms and fresh greenery in a nearby urban park induced physiological and psychological relaxation. This could be developed into an effective park therapy program for middle-aged subjects in the early stages of age-related diseases such as hypertension and for elderly subjects to connect with nature and have social interaction, which contributes to stress relief and mental health improvement. This study’s findings suggest that the Japanese tradition of viewing Cherry blossoms in parks during spring with significant landscape features (e.g., flowers, water, greenery) can be integrated into park therapy programs to improve moods and feelings. On the other hand, maintained woodlands with those landscape features as a view in the early summer can be proposed as a viewing point in which people can relax and feel tranquil from the shade. More studies should elevate local values and consider the accessibility and familiarity for the senior citizens. However, this study has limitations, first among which is the small number of subjects participating in park therapy in each season. It was not easy to find middle-aged participants who did not use blood pressure and heart disease medication and could participate in park therapy experiments for 4.5 h. To supplement these findings, more studies should be conducted using larger samples of middle-aged and elderly residents (more than 12 subjects) in order to draw more reliable and significant results. Second, physiological indices used only measured blood pressure and heart rate. Other physiological indices, such as eye movement and brain blood flow, are necessary for comprehensive findings. Third, this study was conducted in only one seated-viewing scene per season in an urban park. For future studies, more seated-viewing scenes per set of the experiment are required. These limitations must be considered in future research.

## 5. Conclusions

This study investigated the physiological and psychological relaxation effects associated with viewing park landscape. Viewing urban parks in spring and early summer resulted in (1) significantly lower blood pressures, (2) significantly increased vigor–activity in spring, (3) significantly decreased total mood disturbance in spring, and (4) significantly decreased anxiety levels in early summer. These findings could be used for park therapy programs for middle-aged and older adults in urban parks during spring and early summer. The composition of park therapy scenes might be arranged by considering the significant natural elements (e.g., flowers, water bodies, and maintained greenery) as input for therapeutic park design to provide higher value of relaxation benefits. In this way, nearby urban park usage is promoted and lifestyle diseases are prevented.

## Figures and Tables

**Table 1:** Details of the experiment sites.

	Spring	Summer
Experimental Period	March 28–April 3	May 30–June 6
Temperature (°C) (mean ± SD)	Park: 14.4 ± 2.8 City: 13.4 ± 2.9	Park: 26.73 ± 2.64 City: 26.73 ± 1.53
Relative humidity (%) (mean ± SD)	Park: 45.4 ± 18.1 City: 40.6 ± 14.2	Park: 50.2 ± 10 City: 52 ± 5.71

**Table 2:** Participant characteristics.

Parameter	Spring (*N* = 12)	Summer (*N* = 12)
Age (years)	66.4 ± 10.5	66.8 ± 10.1
Gender		
Male	50%	41.7%
Female	50%	58.3%
Employment status		
No	66.7%	75%
Yes	33.3%	25%
Education		
Senior high school	25%	33.3%
University	16.7%	33.3%
Other	58.3%	33.3%
Income (JPY/month)		
Less than JPY 150,000	41.7%	58.3%
JPY 150,000–200,000	25%	25%
JPY 200,000–250,000	16.7%	-
More than JPY 250,000	16.7%	16.7%
Smoking behavior		
No	91.7%	91.7%
Yes	8.3%	8.3%
Alcohol use		
No	58.3%	50%
Yes	41.7%	50%
Sleeping time (hours)		
Less than 7 h	66.7%	66.7%
7–9 h	33.3%	33.3%
Sport activity (times/month)	5 ± 5.5	7.6 ± 9.3
Shinrin-yoku (times)	6.6 ± 5	5.6 ± 8.5
Social attachment		
Sometimes	66.7%	50%
Often	25%	16.7%
Always	8.3%	33.3%
Participation in community (times/month)	1.6 ± 1.9	1.6 ± 2.8

**Table 3:** Time schedules during the viewing experiment of park therapy in spring and early summer. POMS: Profile of Mood States; STAI: State-Trait Anxiety Inventory.

Time	Activities (Location)
08:30	Gathering at meeting point (in front of station building)
08:30–09:00	Dropping off to park/city area by car
09:00–09:20	Orientation and signing of consent forms (resting room)
10:20–10:40	Pre-measurement of blood pressure and heart rate, POMS, STAI (resting room)
10:40–11:00	Seated-viewing experiment (park/city)
11:00–11:20	Post-measurement of blood pressure and heart rate, POMS, STAI (resting room)
11:20–12:00	Moving to the urban park/city by car
12:00–12:30	Having lunch (resting room)
13:20–13:40	Pre-measurement of blood pressure and heart rate, POMS, STAI, (resting room)
13:40–14:00	Seated-viewing experiment (park/city)
14:00–14:20	Post-measurement of blood pressure and heart rate, POMS, STAI (resting room)

**Table 4:** Verification of internal consistency.

Indices	Cronbach’s α	
	Spring	Summer
Heart rate	0.815	0.824
Blood pressure	0.931	0.781
POMS	0.71	0.896
STAI	0.896	0.933
